# Diet-Induced Rodent Models of Diabetic Peripheral Neuropathy, Retinopathy and Nephropathy

**DOI:** 10.3390/nu12010250

**Published:** 2020-01-18

**Authors:** Inês Preguiça, André Alves, Sara Nunes, Pedro Gomes, Rosa Fernandes, Sofia D. Viana, Flávio Reis

**Affiliations:** 1 Institute of Pharmacology & Experimental Therapeutics, & Coimbra Institute for Clinical and Biomedical Research (iCBR), Faculty of Medicine, University of Coimbra, 3000-548 Coimbra, Portugal; i.preguica@campus.fct.unl.pt (I.P.); alves.andrefb@gmail.com (A.A.); sara_nunes20@hotmail.com (S.N.); pgomes70@gmail.com (P.G.); rcfernandes@fmed.uc.pt (R.F.); sofia_viana@estescoimbra.pt (S.D.V.); 2 Center for Innovative Biomedicine and Biotechnology (CIBB), University of Coimbra, 3004-504 Coimbra, Portugal; 3 Department of Biomedicine, Faculty of Medicine, University of Porto, 4200-319 Porto, Portugal; 4 Center for Health Technology and Services Research (CINTESIS), University of Porto, 4200-450 Porto, Portugal; 5 Polytechnic Institute of Coimbra, ESTESC-Coimbra Health School, Pharmacy, 3046-854 Coimbra, Portugal

**Keywords:** type 2 diabetes mellitus, microvascular complications, diabetic peripheral neuropathy, diabetic retinopathy, diabetic nephropathy, rodent models, diet-induced

## Abstract

Unhealthy dietary habits are major modifiable risk factors for the development of type 2 diabetes mellitus, a metabolic disease with increasing prevalence and serious consequences. Microvascular complications of diabetes, namely diabetic peripheral neuropathy (DPN), retinopathy (DR), and nephropathy (DN), are associated with high morbidity rates and a heavy social and economic burden. Currently, available therapeutic options to counter the evolution of diabetic microvascular complications are clearly insufficient, which strongly recommends further research. Animal models are essential tools to dissect the molecular mechanisms underlying disease progression, to unravel new therapeutic targets, as well as to evaluate the efficacy of new drugs and/or novel therapeutic approaches. However, choosing the best animal model is challenging due to the large number of factors that need to be considered. This is particularly relevant for models induced by dietary modifications, which vary markedly in terms of macronutrient composition. In this article, we revisit the rodent models of diet-induced DPN, DR, and DN, critically comparing the main features of these microvascular complications in humans and the criteria for their diagnosis with the parameters that have been used in preclinical research using rodent models, considering the possible need for factors which can accelerate or aggravate these conditions.

## 1. Introduction

Diabetes mellitus is considered one of the primary contributors to non-communicable diseases. Estimates from the International Diabetes Federation (IDF) suggest that the number of persons afflicted by this disease will rise from 463 million in the year of 2019 to 700 million by 2045 [1]. Type 2 diabetes mellitus (T2DM) accounts for roughly 90% of all individuals with diabetes and the expected increased prevalence directly affects neuropathy, retinopathy, and nephropathy burden, collectively alluded as the classical diabetic microvascular complications [2].

T2DM onset and early progression is a silent process; however, at the time of diagnosis, microcirculatory damage is often present with multiorgan consequences [3,4]. The evolution of diabetic microvascular complications is closely linked with longstanding or uncontrolled disease and may ultimately culminate in severe disabilities, such as diabetic foot ulcers, blindness, and end stage renal disease (ESRD), with increased costs for patients and society [5]. Unfortunately, contemporary glucose-lowering medications have been disappointing to halt or slow down diabetic microvascular injury [6]. Thus, updated preclinical approaches are needed to gain new insights on the basic function of diabetic microvasculature and successfully improve unmet therapies [7].

Animal models have been acknowledged for several decades as useful tools to study metabolic disorders. To obviate the gap between preclinical and clinical research, experimental models aimed to replicate diabetic microvasculature dysfunction should ideally emulate T2DM main stressors and mimic the orchestrated mechanisms underlying human diabetes progression [8,9]. It is generally believed that T2DM is driven by a complex interplay of genetic factors and unhealthy lifestyle habits comprising an energy-dense “westernized” diet [10,11,12,13,14]. Hence, diet-induced rodent models of T2DM, whether alone or combined with genetic/chemical stressors, are paramount to more closely replicate human microvascular complications [15,16]. The most commonly used diets for T2DM animal research are high-fat diets (HFD), high-sugar diets (HSD), and Western diets combining both high-fat and high-sugar components (HFSD) [17]. Western diets have been a usual choice due to their ability to replicate human unhealthy dietary patterns along with a more robust and reproducible animal phenotype. Yet, the lack of standardization of content/source of macronutrients as well as protocol duration currently challenges experimental data reproducibility and a fair translation of preclinical data [17].

Herein, we intended to revisit diet-induced rodent models, whether alone or combined with genetic or chemical tools, to successfully replicate the main clinical features underlying classical diabetic microvascular complications.

## 2. Diet-Induced Models of Diabetic Peripheral Neuropathy

Diabetic peripheral neuropathy (DPN) is the most common form of neuropathy worldwide, affecting approximately half of patients with diabetes [18]. It is known to be heterogeneous by its clinical course, symptoms, and pattern of nerve fiber involved, with symmetrical length-dependent sensorimotor polyneuropathy being the prevailing form [19]. A dichotomous phenotype is usually observed since affected patients can experience both negative and positive sensory symptoms, including decreased sensation, numbness and/or pain, motor weakness, impaired proprioception, and gait disturbance [20,21]. A distal-to-proximal axonal degeneration of peripheral large-fibers leads to impairment of vibratory sensation and subsequent numbness along with diminished ankle reflexes. In opposition to this insensate phenotype, a painful symptomatology (e.g., prickling, stabbing, and/or burning sensations) is consistently reported, likely reflecting structural small-fiber damage [22,23].

Besides the dying-back axonal injury, DPN also targets Schwann cells and endoneurial capillaries with inappropriate nerve vascular supply [24,25]. Endotheliopathy in vasa nervorum is therefore a microvascular liability often found in diabetic nerve biopsy samples, paralleling the pathological alterations observed in retinopathy- and nephropathy-related vascular beds [26]. Oxidative stress, inflammation, and advanced glycation end products accumulation that occur secondary to chronic hyperglycemia are some examples of pathophysiological features involved in DPN development [20]. Overall, affected patients can experience a range of complications that may culminate in chronic neuropathic pain and foot ulceration, making DPN the leading cause of non-traumatic amputations in the Western world [27].

Assessment of DPN relies mostly on clinical bedside testing and quantitative sensory assessment that indiscriminately evaluates large and small fiber function through specific devices (e.g., thermoaesthesiometer) able to define subjects’ sensory thresholds. Yet, a more precise phenotyping can be achieved by specialized diagnostic tests. Electrophysiological records of sensory and motor nerve conduction studies (NCS) provide the most objective and noninvasive measure of subclinical peripheral large nerve fiber dysfunction. However, NCS are unable to capture small sensory fiber damage, one of the earliest manifestations of DPN that is markedly observed in the prediabetic condition [20]. Morphometric quantification of intraepidermal nerve fibers (IENF) is considered the gold standard for small fiber neuropathy evaluation, even though the invasive nature of the procedure (skin biopsy) obviates its use in longitudinal and interventional studies. Alternatively, corneal confocal microscopy (CCM) is a more appealing technique as it offers a non-invasive mean to follow corneal sub-basal fibers’ progressive loss [22,28]. Interestingly, it has been recently suggested that CCM fulfills the Food and Drug Administration (FDA) criteria as a valid surrogate endpoint of DPN [28]. Taking into account the aforementioned possibilities for disease assessment, a broad consensus on DPN’s diagnostic criteria was provided by the Toronto diabetic neuropathy expert group, who suggested the use of abnormal NCS with a clinical symptom or sign as the minimal criteria for DPN diagnosis confirmation [22].

**Figure 1:**
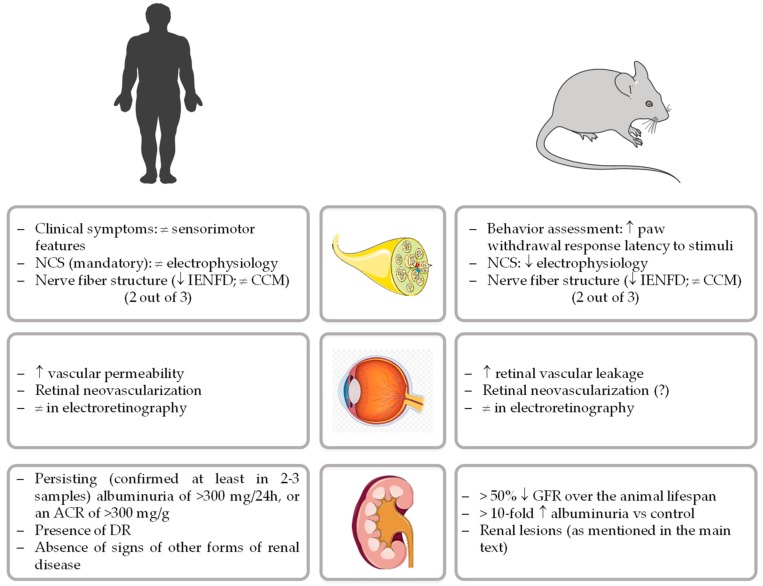
Comparative criteria for human and rodent models of diabetic peripheral neuropathy, retinopathy, and nephropathy. (NCS, nerve conduction studies; IENFD, intraepidermal nerve fibers density; CCM, corneal confocal microscopy; ACR, albumin-to-creatinine ratio; DR, diabetic retinopathy; GFR, glomerular filtration rate).

Currently, and apart from the management of painful neuropathic symptoms, there are no approved disease-modifying therapies for DPN [28]. Systematic failures of new therapeutic opportunities highlight the need for rodent animal models that closely recapitulate the human disorder, a prime experimental scenario for the development and testing of disease-based pharmacological options [19]. To properly advance of this research field, the diabetic neuropathy study group of the European Association for the Study of Diabetes (Neurodiab) proposed a unifying framework to phenotyping rodent models of DPN. The presence of impairments in two out of three assessments of key features present in human pathology (behavior, nerve conductions velocities, and/or nerve structure) was therefore defined as the minimum criteria to establish neuropathy in diabetic rodents [29], as represented in Figure 1.

Genetic animal models of T2DM/DPN (e.g., Bio-Breeding Zucker diabetic rat (BBZDR) /Wor rat, Zucker diabetic fatty (ZDF) rat, Goto Kakizaki (GK) rat, db/db mice, ob/ob mice, diabetic black and tan, brachyuric (BTBR) ob/ob mouse) as well as the streptozotocin (STZ) diabetic rodents (40–80 mg STZ/kg body weight, i.p./i.v.) have been available for years and used to study DPN [30,31,32,33,34,35]. Yet, none follow the gradual onset of metabolic imbalances and the natural progression from obesity to T2DM. Growing literature links increased dietary fat/dyslipidemia to neuropathy development in both metabolic syndrome (MetS)/prediabetic/diabetic patients as well as in experimental rodents [36,37,38,39,40]. Interestingly, Sullivan and colleagues have lent support to this assumption, showing that C57BL/6 db/db mice fed with HFD (17% kcal from fat) presented an exacerbated hyperglycemic and neuropathic phenotype [41]. Hence, animal models of diet-induced obesity (DIO) based on purified high fat or high fat/sugar diets (alone or combined with STZ-induced diabetic conditions) have been broadly used to replicate experimental DPN, as summarized in Table 1.

Typically, chronic HFD/HFSD paradigms are key determinants for the progressive manifestation of behavioral and NCV’s deficits as well as nerve structural impairments [21,44]. In fact, adult C57BL/6J mice fed a HFSD (24% from fat, 41% from carbohydrates (CH)) for 12 weeks display glucose intolerance as well as impairments in aforementioned neurological end-points, including behavioral (hindpaw thermal nociceptive response), NCV’s (motor sciatic-posterior tibial nerve conduction and sensory digital nerve conduction velocities), as well as anatomic IENFD loss (immunoreactive hindpaw IEFND profile) [42,46]. Consistently, adult Sprague-Dawley (SD) female rats submitted to a HFSD (60% kcal from fat, 20% kcal from CH) for 12 weeks presented impaired mechanical allodynia and thermal nociception as well as decreased IENFD and subepithelial corneal nerves. Nevertheless, when hyperglycemia was induced in female obese rats (30 mg/kg STZ, i.p.), sensory nerve density of the skin and cornea as well as thermal and mechanical sensitivity were more significantly impaired, likely reflecting the progressive nature of DPN evolution [44]. It is worth emphasizing the distinct sensitivity to pain between male and female rodents [47], as well as the effect of dietary salt supplementation on DPN hallmarks [45]. Interestingly, male SD albino rats submitted to a high salt/fat/carbohydrate diet (HFSSD: 48% kcal from fat, 37% kcal from CH and 80 g dietary salt/kg diet) for about 16 weeks displayed an aggravated breakdown of large myelin fibers in sciatic nerves when compared with HFSD-fed animals. This observation further reinforces the importance of dietary components in animal models aimed to recapitulate early stages of DPN pathology. Consistently, nutrition-induced diabetic neuropathy (60% kcal from fat, 20% kcal from CH) has been described in 5-week-old C57BL/6J mice as a tool to replicate juvenile DPN [43].

Importantly, the neuropathic phenotype and metabolic impairments observed in C57BL/6 mice fed a HFD (54% kcal from fat) from 4 to 20 weeks were completely normalized by switching standard chow for 4 weeks, highlighting the importance of a short-term dietary reversal paradigm as a disease-modifying treatment for early neuropathy [48].

Overall, diet-induced experimental DPN is a valuable approach to model prediabetes/obesity/MetS-related neuropathy. Yet, alternative genetically and/or chemical-induced approaches, whether alone or in combination with dietary interventions, should be considered when chronic DPN paralleling T2DM evolution is envisaged.

## 3. Diet-Induced Models of Diabetic Retinopathy

DR is a diabetes-specific microvascular complication and remains the leading cause of vision loss in middle-aged and economically active people, in developed countries [49]. Over a third of diabetic patients have signs of DR, and a third of these have vision-threatening DR, which includes severe retinopathy and macular edema [49]. Its prevalence is higher in patients with Type 1 Diabetes Mellitus (T1DM) than in T2DM, and around 25% of patients with the former start to develop the retinal disease within 5 years after diabetes onset, while T2DM patients will have some form of DR after 20 years of the disease [50].

Early stages of DR may be detected by abnormalities in retinal blood flow, changes in retinal vessel caliber, and changes in electroretinography [51,52]. However, clinical diagnosis is based mainly on the presence of fundus features detected by ophthalmoscopic examination. The initial changes detected include the presence of microaneurysms, dot and blot hemorrhages, venous beading, hard exudates, and cotton wool spots. Microaneurysms formation is associated with loss of supporting pericytes and/or glial attachment, and alterations of the capillary basement membrane [53]. DR progresses, from mild non-proliferative form, characterized by increased vascular permeability, to a moderate and severe non-proliferative phenotype, depicted by several regions of capillary closure which lead to retinal ischemia and induce upregulation of pro-inflammatory cytokines and angiogenic factors. DR can even progress to a more severe stage, the proliferative DR, characterized by the growth of new and leaky blood vessels (neovascularization) on the retina and posterior surface of the vitreous [54]. Diabetic macular edema (DME), characterized by retinal thickening resulting from blood retinal barrier breakdown, often occurring in association with altered homeostasis in Muller cells, can develop in all stages of DR and it represents the most common cause of vision loss in patients with diabetes [54,55].The pathogenesis and development of DR are highly complex due to the participation of multiple interlinked mechanisms that lead to cell injury and cellular adaptive changes in the retina [56]. It is increasingly evident that there are functional and structural changes in microvascular and neuroglial components, but the exact underlying mechanisms remain to be completely defined.

Rodents have been extensively used as models to better understand the etiology and pathogenesis of DR and of great value in providing insight into efficient and effective therapy development. A major criticism of using rodents is that they may not always perfectly mimic the human DR, namely the proliferative stage of the disease, which is characterized by neovascularization and subsequently, retinal detachment. Although there are some reports showing the presence of retinal neovascularization in rodents, this remains controversial, at least, in some animal models, due to differences in the detection methods applied, strains and age.

Although no single animal model fully mimics all the neuroglial and vascular changes that are present at each stage of DR, as well as the entire pathophysiological progression observed in humans, its use provides insights into the molecular and cellular basis of DR. During the last decades, several genetic rodent models were developed to recapitulate some features of human clinical DR and to test new therapeutic agents [57,58]. Despite the fact that transgenic animals overexpressing vascular endothelial growth factor (VEGF) in retinal cells can present the key features of proliferative DR, such as neovascularization, these models can neither recapitulate the metabolic changes due to prolonged hyperglycemia of diabetes nor accurately imitate DR progression. Therefore, these models should be avoided in studies with the purpose of addressing research questions associated with DR etiology or development of preretinal neovascularization, in which new blood vessels invade the vitreous.

Although HFD feeding protocols are commonly used to induce obesity in association with early T2DM conditions in rodents, such as insulin resistance (IR), in most of the cases, they only develop the retinal microvascular lesions observed in human DR after extended periods of HFD consumption, therefore being a slow onset disease model. In fact, C57BL/6 mice fed HFD (for 16–20 weeks), in which obesity is present, were shown to present retinal ganglion cell (RGC) dysfunction (measured by the response of the retina to patterned light stimuli—PERG). However, HFD (42%–45% kcal from fat) for 24 weeks failed to induce the increased retinal vascular permeability, one of the main features of early stages of DR, which only occurs after 48 weeks of HFD feeding [59,60] (Table 2), suggesting that in a diabetes model induced by HFD feeding, the electrophysiological abnormalities of RGCs precede the development of visible retinal microvascular damage. Consistently, in another study, the same mouse strain (C57BL/6J) fed HFD (59.5% kcal from fat) for 1 month developed mildly compromised retinal light responses, with decreased oscillatory potential (OP) and delayed OP implicit times), even before systemic hyperglycemia was installed, suggesting that neural retina dysfunction may precede systemic hyperglycemia. The authors postulated that HFD-induced obesity might have a negative effect on eyesight even before the diagnosis of diabetes. However, this should be confirmed with clinical research correlating human dietary habits and vision impairment. In another study, using a different mouse strain, the Swiss mice fed HFD for 8 weeks was shown to be sufficient to induce an increased body weight, increased fasting glucose, decreased insulin tolerance, and decreased OP (but without differences among amplitudes of wave a, b, or c, which are responsive for cones, rods, and epithelial cells, respectively). However, after 16 weeks, HFD induced a pro-inflammatory state in the retinal tissue (increased tumor necrosis factor (TNF)-α and interleukin 1 beta (IL-1β) gene expression and protein levels) and significantly increased levels of the inducer of VEGF that regulates angiogenesis by inducing proliferation of retinal endothelial cells and enhances vascular permeability [61].

A rodent model using HFD combined with STZ injection (single injection of high dose or multiple injections of low dose) to induce chronic hyperglycemia is the most widely accepted animal model for studying retinal complications in T2DM [8,62,64]. This model manifests metabolic abnormalities in body weight, plasma glucose and lipid levels, liver, and renal function [62]. Depending on the injection protocol in terms of dosage and number of injections, rodent types, age, and genetic background of the animals, retinal abnormalities manifest in different ways. For instance, C57BL/6J mice fed a HFD for 12 weeks and then intraperitoneally injected with a low dose of STZ (30 mg/kg) for 7 consecutive days, and maintained for more 12 weeks, were shown to present main features of early stages of DR, such as loss of pericytes and acellular capillaries formation, increased retinal vascular leakage, increased oxidative stress (increased reactive oxygen species (ROS) production and nicotinamide adenine dinucleotide phosphate oxidase 2 (NOX2) expression, accompanied by a decrease of an important antioxidant, superoxide dismutase 2 (SOD2)), chronic retinal inflammation (increased TNF, IL-1β and VEGF), and increased apoptosis [62]. SD rats are also sensitive to HFD feeding in combination with only one STZ injection of the same dosage (30 mg/kg). In fact, after 16 weeks of treatment, diabetic SD rats presented some features of early stages of DR, such as abnormal b-waves and OPs, and diabetic rats had lower total retina and outer nuclear layer (ONL) thickness compared to control rats [63].

A protocol that combines the main diet stressors encountered in the human population, HFD combined with high sugar, such as fructose [8], for a longer period can be useful in rodent studies that aim to reproduce the pathological progression of human DR. Wistar rats fed a high fat and fructose diet (HFFD) for 56 weeks and intraperitoneally injected with low-dose STZ multiple times can recapitulate certain features of diet-induced T2DM seen in humans, from the initial phase (2–18 weeks), characterized by moderate glucose intolerance and impaired glucose metabolism associated to IR, followed by a transition from pre-diabetes and or IR to frank T2DM, characterized by a significant fasting and post IPGTT hyperglycemia, dyslipidemia, fructosamine and glycated hemoglobin, pancreatic beta cell dysfunction, insulinopenia, and decrease in body weight gain. As early as week 20, the diabetic animals recapitulate long-term complications associated with the development of T2DM, such as the presence of retinal morphological lesions, namely the thickening of the retinal parenchyma and pathological neovascularization [8].

The Zucker fatty/Spontaneously hypertensive heart failure F1 hybrid (ZSF1) rat, a spontaneous diabetic model with hypertension and obesity which is commonly used as a DN model, are bred by crossing lean female Zucker diabetic fatty (ZDF) rats with lean male spontaneously hypertensive heart failure rats (SHHRs), thus creating a model with many features of the human T2DM [65,66]. However, it seems that there is a lack of retina damage in these rats [67], in contrast with the impact on other organs targeted by microvascular complications of diabetes, namely the kidney [65,66]. In fact, in obese 42 weeks old ZSF1 rats, the vascular density pericyte coverage, microglia number, vascular morphology, and retinal thickness are not affected by chronic hyperglycemia, which suggest that these rats do not develop DR. Although it is not a suitable model of DR, ZSF1 rats can be a useful model to identify key molecules and better understand their protective role in the retina [67].

## 4. Diet-Induced Models of Diabetic Nephropathy

T2DM is the most common cause of chronic kidney disease (CKD) and ESRD worldwide [68]. DN, a long-term major microvascular complication of T1DM and T2DM, affects a large population in the United States and Western Europe, where around one-third of diabetic individuals have nephropathy [69].

DN is typically characterized by an initial stage of glomerular hypertrophy, moderate expansion of the mesangial matrix, and thickening of the glomerular capillary walls. As the disease progresses, glomerulosclerosis is the main feature, as a result of thickening of the glomerular basement membrane (GBM), mesangial cell expansion and loss of podocyte, followed by tubulointerstitial fibrosis, accompanied by progressive albuminuria, reduction in glomerular filtration rate (GFR), fluid retention, and elevation of blood pressure [70]. DN is also characterized by an increased urinary albumin excretion (UAE) and is divided into micro and macro albuminuria [71,72]. However, the pathophysiological mechanisms underlying DN development remain to be completely elucidated.

The relevance of animal models, particularly rodents, for the study of the complex pathogenesis of DN and to evaluate the impact of therapeutics is unequivocal [73]. Ideally, an animal model for DN should present many features resembling humans, especially similar kidney anatomy and physiology, as well as the ability to allow determination of hemodynamic and biochemical parameters throughout a chronic stable pathophysiological evolution. However, despite the major progress made to develop good animal models of DN, none of them are able to completely recapitulate the functional and structural changes of an established human DN [74]. Aiming to develop and phenotype new animal models more closely resembling the human disease, the National Institute of Diabetes and Digestive and Kidney Diseases (NIDDK) created, in 2003, The Animal Models of Diabetic Complications Consortium (AMDCC), that proposed the following 3 criteria for a desirable murine model of DN [75]: (1) more than 50% decline in GFR over the lifespan of the animal, (2) greater than 10-fold increase in albuminuria compared with controls for that strain at the same age and gender, and (3) histopathology findings which include mesangial sclerosis (a 50% increase in mesangial volume), any degree of arteriolar hyalinosis, GBM thickening (a >25% increase compared with baseline by electron microscopy morphometry), and tubulointerstitial fibrosis (Figure 1).

Although there are no murine models that meet all 3 of the criteria, there are some genetic models that recapitulate important features of the human disease [76,77,78]. Independently of the ability of genetic engineering to improve the quality of models, diet manipulation could be a major contribution to create a stage of DN better resembling the human features. In fact, there are several models of DN that benefit from use of hypercaloric (typically high-fat and/or high-sugar) diets, the majority of them in combination with other drivers of disease evolution, such as the use of: (i) STZ to promote a desirable degree a beta-cell decline, (ii) genetic models, namely the obese ZSF1 rat, or (iii) nephrectomy strategies to reduce kidney mass and increase the renal decline and lesions.

HFD-based protocols are widely used to induce metabolic impairment, including IR and obesity, being useful to DN research (Table 3). However, inbred strains of mice present significant differences in response to HFD: while A/J mice are relatively resistant, C57BL6 mice are highly responsive to HFD [79]. In fact, HFD-fed C57BL/6 mice develop features resembling human metabolic syndrome, such as obesity, hyperglycemia, hyperinsulinemia, hypertriglyceridemia, and hypertension, as well as increased UAE and renal glomerular lesions associated with extracellular matrix protein accumulation, together with impaired sodium handling, lipid accumulation, macrophage infiltration, and oxidative stress [80]. On the contrary, HFD-fed Wistar rats, even for a long period (20 to 28 weeks), are unable to develop major changes on renal function and basal microvascular blood flow, despite presenting kidney endothelial dysfunction and markers of increased renal oxidative stress and inflammation [81].

In order to overcome the absence of some of the main structural features of human DN seen in the models with HFD, researchers have been using HFD accompanied by a single high-dose STZ to aggravate pancreatic damage and induce a more advanced diabetic kidney disease. However, there are several limitations of this model of T1DM, as recently discussed [82]: (i) since animals are insulinopenic, insulin replacement is required but there is no consensus on the most adequate regimen (if used at all) and glycemic control obtained, thus causing a major disparity of results, (ii) a single, high dose of STZ (typically between 55 to 65 mg/kg) is needed, which causes nephrotoxicity, and (iii) although relevant renal lesions are replicated in this model, namely thickening of GBM, mild expansion of the mesangial matrix, and tubulointerstitial alterations in some rat species, glomerulosclerosis (diffuse and/or nodular), typical of human DN, is absent.

Unlike high-dose STZ, which induces T1DM, rats submitted to a combination of HFD and low-dose STZ present a moderate degree of pancreatic damage and glucose intolerance, similar to what is found in early stages of T2DM. In addition, despite lower blood glucose levels and proteinuria, animals will develop more severe kidney lesions [82]. This protocol has been also used in combination with different degrees of kidney mass removal by nephrectomy. In heminephrectomized rats, the use of a single low dose of STZ (40 mg/kg, i.v.) followed by HFD feeding, will cause major metabolic changes between 15 and 25 weeks, including hyperglycemia and reduced plasma insulin, accompanied by hyperlipidemia and hypertension [92]. An identical model was reported by Sugano et al. [83] using HFD and multiple (9 consecutive days) low-dose (40 mg/kg) injections of STZ in uninephrectomized rats. When aged 15 weeks, these animals present microalbuminuria and augmented creatinine clearance, which were followed (at the age of 25 weeks) by overt proteinuria, mesangial expansion, and terminal glomerular sclerosis (Table 3).

The STZ + HFD models are responsive to dietary and/or pharmacological interventions able to normalize hyperglycemia, hyperlipidemia, or hypertension, thus being useful tools to test whether different treatment modalities could or could not retard the progression of DN. Although albuminuria and several early renal lesions are manifest in these models (such as thickening of GBM and mesangial expansion), some relevant histopathologic features of advanced stages of disease (such as arteriolar hyalinosis and nodular glomerulosclerosis) are absent or inconsistent.

Most animal models, particularly the mouse ones, despite presenting albuminuria/proteinuria and glomerular lesions, do not manifest tubulointerstitial fibrosis and do not develop a progressive renal function failure. In an effort to develop new models of DN, several researchers, including those of the AMDCC, have been using genetic breeding and other tools to aggravate disease severity, in such a way that the main features of DN are becoming more evident. Some of the new models are obtained by combinations of genetic models with hypercaloric diets, such as the diabetic obese ZSF1 rats. Both lean and obese ZSF1 rats have high blood pressure, but only the obese animals (fed a high carbohydrate diet) are able to develop hyperglycemia, hyperlipidemia, and renal dysfunction [65,66]. When aged 20 weeks, obese ZSF1 rats showed renal lesion resembling early DN, such as arteriolar thickening, tubular dilation and atrophy, thickening of GBM, and mesangial expansion [84]. This model demonstrates the progression of several major features of type 2 DN, namely obesity, hyperglycemia, hyperlipidemia, and renal function decline, which is accompanied by proteinuria and kidney glomerular and tubulointerstitial fibrosis, which could be accelerated by uninephrectomy [85]. A key aspect of the ZSF1 model that differentiates it from other rodent models of DN, particularly in mice, is the development of significant tubulointerstitial fibrosis, a key feature of human DN. Although ZDF rats are able to develop DN, as well as DR [93,94,95], obese ZSF1 rats develop progressive aggravation of renal disease with death accompanied by ESRD when aged 45–50 weeks [65,66,86], unlike the parental backgrounds from which they are derived (ZDF and SHHF rats).

Finally, fructose used to be considered beneficial as an energy source for diabetic patients but epidemiological studies have suggested that excessive fructose intake can contribute to obesity, diabetes, hypertension, and kidney disease, namely by raising uric acid [96,97]. The American Diabetes Association made a proposal not to recommend fructose supplementation for diabetic subjects [98].

There is evidence that soft drinks containing high amount of fructose can raise albuminuria in humans and increase the risk for renal disease [99]. In animal studies, a high-fructose diet promotes metabolic impairment, elevation of blood pressure, and hyperuricemia in normal rats and exacerbates complications in diabetic models, at least in part by uric acid formation [100]. A high-fructose diet has been associated with development of glomerular hypertension, renal microvascular damage, and tubulointerstitial injury in normal and diabetic rats [87,88,91]. Fructose-induced kidney injury was compared in 3 mouse strains (C57Bl/6J, CBA/JN and DBA/2N) and only the DBA/2N mice was able to develop tubulointerstitial fibrosis [91], which seems to be linked with increased expression of GLUT5. Furthermore, a parallel study confirmed the HFFD C57BL/6 as a poor model for the study of DN [101]. However, a recent study showed that a high-fructose diet can accelerate DN in the Spontaneously Diabetic Torii (SDT) rat [89]. The SDT rat is a non-obese T2DM model that when aged about 24 weeks old, develops renal lesions accompanied by increases in urine volume and renal function parameters, which progresses with aging to severe tubular lesions, diffuse, and nodular glomerular lesions [90].

## 5. Concluding Remarks

The growing incidence of patients presenting diabetic microvascular complications is in line with the alarming increasing prevalence of T2DM in many world regions. Diabetic neuropathy, retinopathy, and nephropathy are strongly associated with increased morbidity and reduced life quality of patients and of their families, also contributing to a reduced availability to work, altogether imposing tremendous socioeconomic and medical costs.

Despite major efforts to develop more efficient strategies to prevent or at least to halt progression of diabetic microvascular complication [102], the available options are clearly limited and the battle is still lost. The conclusion has two facets: (i) we were still unable to discover the main mechanisms involved, the most promising targets, and/or the most effective therapeutic tools, although we are using the right models, techniques, and approaches regarding the current scientific knowledge, or (ii) we are not even using the most appropriate weapons, so success can only happen by chance. Perhaps both conclusions can coexist, and we need to move forward in both ways and converge in the right direction. The complexity of each of these diseases/complications (including the high number of risk factors involved and the multiple coexisting pathophysiological mechanisms), the nebulous definition of precise diagnostic criteria (namely for humans), as well as the difficulty of make use of human samples, greatly complicates this mission.

In light of this, animal models can be a valid instrument, particularly if they can reproduce the main features of those diseases, replicate their evolution, and if they are being used with criterion and standardization by different institutions and researchers. In order to promote the standardization needed, groups and/or organizations have proposed unifying frameworks to phenotyping rodent models of diabetic microvascular complications, namely for neuropathy [29] and nephropathy [75], taking into consideration the human diagnosis criteria, as summarized in Figure 1. In spite of that, over the past few years, as the use of animal models has become widespread, there is some disappointment with the difficulty of choosing a good model and of being able to replicate the results of other laboratories, even using similar experimental settings.

Distinct animal models could be developed with diverse induction tools, including genetic, chemical, and dietary manipulation. Although there are no perfect models, the use of hypercaloric diets as a way to induce the disease is a relevant strategy since this is one of the main risk factors for the onset and evolution of diabetes and its complications; in addition, it is associated with a slow evolution, mimicking the human disease and allowing to test the effectiveness of preventive or curative strategies. However, there are several aspects to consider when using a diet-induced model. First of all, it is particularly difficult and time-consuming to get overt diabetes only with hypercaloric diets, especially in rats, and it is often necessary to promote aggravation with other approaches, namely the combination of high-fat and high-sugar diets and promotion of pancreatic injury using a toxic chemical agent, the most popular being STZ. For the development of more consolidated stages of neuropathy, retinopathy, and nephropathy, this is a fundamental approach, as clearly described in this article [8,41,83].

In addition, a number of other factors that may interfere with the model must be taken into account, including diet, animal species and strain, gender, and age. As discussed throughout the article, there are a variety of diets (with different components and percentages) that promote distinct degrees of dysfunction and lesion, due to distinct impact on the metabolic pathways underlying disease appearance and evolution, as it is recognized that diabetic complications are affected by various factors, including obesity, IR, hyperglycemia, and hyperlipidemia. Additionally, it is known that there are large variations in sensitivity/resistance to hypercaloric diets between different species (a paramount example is the C57BL/6 mice versus the Wistar rat when fed with HFD [80,81]) and even for different strains of the same species [79,91,103], namely, regarding sensitivity to the development of distinct complications, as reported for retinopathy and nephropathy in the ZSF1 rat under HFD [67,85]. Age is also a bias factor, as diabetic microvascular complications may have a distinct phenotype depending on the animals’ age. The same is also true for gender, and it is now clearly accepted that studies of metabolic diseases, among others, should be carried out on individuals of both genders.

In conclusion, diet-induced rodent models exhibit the main features of human DPN, DR, and DN, as well as different disease stages, namely when done using combined diets and in the presence of a low-dose STZ. These models may be crucial for studying the pathophysiology of these complications, as well as to identify new targets and test therapeutic approaches. However, if we are to effectively evolve knowledge and be able to intervene in the evolution of disease, we must be highly judicious when choosing the experimental animal design.

## Figures and Tables

**Table 1:** Main features of diet-induced rodent models of diabetic peripheral neuropathy.

Diet Type	Composition (%)	Duration (Wks)	Species/Strain	Main Features of DPN Evolution	Ref.
HFD	45% calories from fat	34	Mice/C57BL/6J	Thermal hypoalgesia (week 12, 34); Motor and sensory nerve conduction abnormalities (week 12, 34); Decreased IENFD (week 34)	[39]
	45% calories from fat	12	Mice/C57BL/6J	Thermal hypoalgesia (week 6); Sensory nerve conduction abnormalities (week 16, 24 and 36); Decreased IENFD (week 12)	[42]
	60% calories from fat	31	Mice/C57BL/6J	Thermal hypoalgesia (week 24 and 36); Motor and sensory nerve conduction abnormalities (week 16, 24 and 36); Decreased IENFD (week 36)	[43]
	60% calories from fat	12	Rat/Sprague-Dawley	Thermal hypoalgesia and mechanical allodynia (week 12); Sensory nerve conduction abnormalities (week 12); Decreased IENFD and corneal nerve fiber length (week 12)	[44]
HFD + moderate dose STZ	60% calories from fat	31	Mice/C57BL/6J	Thermal hypoalgesia (week 24 and 36); Motor and sensory nerve conduction abnormalities (week 16, 24 and 36); Decreased IENFD (week 36)	[43]
HFD + low dose STZ	60% calories from fat	12	Rat/Sprague-Dawley	Thermal hypoalgesia and mechanical allodynia (week 12); Motor and sensory nerve conduction abnormalities (week 12); Decreased IENFD and corneal nerve fiber length (week 12)	[41]
HFSD	46% calories from fat, 36% calories from CH	16	Rat/Sprague-Dawley	Reduced paw withdrawal mechanical threshold and thermal latency (week 8); Motor coordination impairments (week 16); Severe damage of large myelinated fibers along with mild damage to small myelinated and unmyelinated fibers (week 16)	[45]

CH: carbohydrates; HFD: high fat diet; HFSD: high-fat and high-sugar diet; DPN: diabetic peripheral neuropathy; IENFD: Intraepidermal nerve fiber density; STZ: streptozotocin.

**Table 2:** Main features of diet-induced rodent models of diabetic retinopathy.

Diet Type	Composition (%)	Duration (Weeks)	Specie/Strain	Main Features of DR Evolution	Ref.
HFD	45% calories from fat, 35% calories from CH	16 to 24	Mice/C57BL/6	Electrophysiological impairment of retinal ganglion cells, including delayed and diminished visual responses to patterned light stimuli, precede increased retinal vascular permeability	[59]
	42% calories from fat, 43% calories from CH	24 to 48	Mice/C57BL/6J	Accelerated weight gain and adiposity, with abnormal glucose metabolism and electroretinographic dysfunction manifested by increased latencies and reduced amplitudes of OP (6 weeks); evidence of microvascular disease (48 weeks)	[60]
	35% calories from fat	16	Mice/Swiss	Pro-inflammatory state in the retinal tissue; Increased VEGF protein levels	[61]
HFD + low dose STZ	17.9% calories from fat	12	Mice/C57BL/6J	Pericytes dropout, acellular capillaries formation, increased vascular leakage, increased oxidative stress, chronic retinal inflammation and increased apoptosis	[62]
	10% calories from fat	16	Rat/Sprague–Dawley	ERG abnormalities (abnormal b-wave amplitudes and OPs); Thinner ONLs and fewer cells in the ONLs of the retinas	[63]
HFD + high fructose	25.7% calories from fat; 46.5 wt% fructose	20	Rat/Wistar	Thickening of the retinal parenchyma and neovascularization	[8]

CH: carbohydrates; DR: diabetic retinopathy; ERG: electroretinogram; HFD: high fat diet; ONLs: outer nuclear layers; OP: oscillatory potential; STZ: streptozotocin; VEGF: vascular endothelial growth factor.

**Table 3:** Main features of diet-induced rodent models of diabetic nephropathy.

Diet Type	Composition (%)	Duration (Weeks)	Species/Strain	Main Features of DN Evolution	Ref.
HFD	30% calories from fat	20 to 28	Rat/Wistar	Unable to develop major changes on renal function and basal microvascular blood flow	[81]
	60% calories from fat	12	Mice/C57BL/6	Increased UAE and glomerular lesions associated with extracellular matrix protein accumulation, together with impaired sodium handling	[80]
HFD + low dose STZ	60% calories from fat	5	Rat/Sprague–Dawley	Despite lower blood glucose levels and proteinuria, animals will develop more severe kidney lesions	[82]
	11.3% calories from fat	15 to 25	Rat/Sprague–Dawley	Microalbuminuria and augmented creatinine clearance, followed (25 weeks) by overt proteinuria, mesangial expansion and glomerular sclerosis	[83]
High-CH	56% calories from CH	20	Rat/Obese ZSF1	Renal lesion resembling early DN, such as arteriolar thickening, tubular dilation and atrophy, thickening of GBM and mesangial expansion, with proteinuria and tubulointerstitial fibrosis.	[65,66,84,85,86]
		45–50		Progressive aggravation of renal disease with death accompanied by ESRD when aged 45–50 weeks	
High-fructose	6o% calories from fructose	6 to 8	Rat/Sprague–Dawley	Renal hypertrophy, arteriolopathy, glomerular hypertension, and cortical vasoconstriction, together with cell proliferation and hyperplasia in proximal tubules (focal tubulointerstitial injury)	[87,88]
	66% calories from fructose	4	Rat/SDT	Renal lesions accompanied by increases in urine volume and renal function parameters, which progresses with aging to severe tubular lesions, diffuse and nodular glomerular lesions	[89,90]
	67% calories from fructose	16	Mice DBA/2N	Kidney injury was compared in C57Bl/6J, CBA/JN and DBA/2N, and only the DBA/2N mice was able to develop tubulointerstitial fibrosis	[91]

CH: carbohydrates; DN: diabetic nephropathy; HFD: high fat diet; ESRD: end-stage renal disease; GBM: glomerular basement membrane; UAE: urinary albumin excretion.
